# A Bibliometric Analysis of 30 Years of Pediatric Surgery Publications in Saudi Arabia

**DOI:** 10.7759/cureus.91476

**Published:** 2025-09-02

**Authors:** Raif Nassir, Ghadi Askar, Abdulrahman M Nasser, Nader Ashraf, Hassan Shah, Norah Alsabty, Tariq Alfadda

**Affiliations:** 1 Pediatric Surgery, King Salman Medical City, Madinah, SAU; 2 Pediatrics, Al Jalila Children's Speciality Hospital, Dubai, ARE; 3 Internal Medicine, Prince Mohammed Bin Abdulaziz National Guard Hospital, Madinah, SAU; 4 Clerkship, King Faisal Specialist Hospital and Research Centre, Riyadh, SAU; 5 Medicine and Surgery, Alfaisal University College of Medicine, Riyadh, SAU; 6 Medicine, Alfaisal University College of Medicine, Riyadh, SAU; 7 Pediatric Surgery, King Abdulaziz Medical City, Riyadh, SAU; 8 Pediatric Surgery, King Fahad Medical City, Riyadh, SAU

**Keywords:** bibliometric analyses, citation analysis, clinical research productivity, healthcare research, kingdom of saudi arabia (ksa), pediatric surgery

## Abstract

Pediatric surgery is a relatively new specialty in Saudi Arabia. Over the years, the number of specialized institutions and pediatric surgeons has gradually increased. This study aimed to analyze the quantity, quality, and structural indicators of pediatric surgery publications in Saudi Arabia. A retrospective online review of pediatric surgery publications affiliated with Saudi institutions was conducted from January 1991 to December 2020.

A total of 496 publications were analyzed. The annual number of publications increased over time, peaking at 35 in 2014. The highest cumulative number of publications over five years was 131 between 2016 and 2020. Most publications (65.7%) were affiliated with the Ministry of Health and the Ministry of Education. Regionally, the majority were from institutions in Riyadh, followed by Makkah, the Eastern Region, and Asir. Case reports and case series constituted 49.4% of the publications, followed by retrospective chart reviews (30.2%). Notably, 96.3% of the publications were unfunded, and interinstitutional collaboration was limited (5.6%). The highest citation count was 179, with a peak age-corrected citation rate of 19.89 per year. While the number of publications increased, citation rates declined after peaking between 2006 and 2010.

Based on our findings, although pediatric surgery research productivity is increasing, most publications are case reports or case series. Limited collaboration and funding may influence research quality and citation impact.

## Introduction

Bibliometric analysis is an evolving field of science that applies mathematical and statistical measures to describe, evaluate, or compare publications in a specific field [1]. The number of bibliometric publications has increased significantly in recent decades [1]. The applications of bibliometric analysis in the medical sciences include examining the quantitative and qualitative parameters of authors, journals, institutions, or specialties; comparing publications; and evaluating trends [2]. Bibliometrics are also utilized by governmental sectors or grant committees to aid their decision-making; they provide crucial information to scholars, policymakers, and clinicians [2,3]. Bibliometric indicators are categorized into three types: quantity, performance, and structural indicators. Quantity indicators evaluate productivity, performance indicators assess the quality of scholarly works, and structural indicators evaluate the connection between publications, authors, and research areas [4]. The discipline of bibliometric studies remains unexploited with numerous applications, and the surge in bibliometric-related publications reflects the potential benefits of these studies in different medical and academic fields [1,2].

In pediatric surgery, multiple bibliometric studies have been conducted, including assessments of fields of research, research journal output, countries’ contributions to literature, and training program productivity [3,5-9]. The evolution of the European Journal of Pediatric Surgery, a leading journal in the field, since its establishment over 40 years ago was studied focusing on the structure of the journal, the gradual change in language from Dutch, German, and other European languages to English over time and the transformation to an international journal that had a tangible effect on pediatric surgery literature [5]. Publishing trends were studied by comparing journals in pediatric surgery, which showed a significant increase over 30 years, with an increased number of authors, the highest in North America, accounting for 42% of publications [7]. Publications in pediatric surgery have increased by 19% per decade in the last three decades; despite this increase, there has been a noticeable decrease in formerly scientifically active countries [7,8].

In Saudi Arabia, the pediatric surgery specialty was recognized in 1980, and since then, the number of practicing pediatric surgeons has increased to 50 in 20 years, reaching 147 practicing consultants in 2021 [10,11]. The earliest online publications in the field of pediatric surgery from Saudi Arabia were two articles published in 1987 [12,13]. While many national bibliometric studies have been conducted in multiple medical and surgical fields, none have been conducted in the field of pediatric surgery [14-24]. This study aims to assess Saudi Arabia’s contribution to the field of pediatric surgery literature over more than 30 years. Specifically, it analyzes three key aspects: the quantity, quality, and structural characteristics of pediatric surgery publications originating from Saudi Arabia, to help understand the extent and impact of the research from this country in this field.

## Materials and methods

A retrospective online review of the following databases was conducted via convenience sampling: Web of Science (WoS), Scopus, Google Scholar, Embase, and PubMed to retrieve publications from Saudi Arabia. Publications that were affiliated with pediatric surgery and/or authored by a pediatric surgeon in Saudi Arabia from January 1991 to December 2020 were included. Publications that were conducted completely abroad, those that were non-relevant to the pediatric surgery field (except publications in medical education and basic sciences) were excluded. The included publications were research-based only; thus, posters, videos, letters to editors, and book chapters were excluded.

The publications were then examined for data collection. The collected variables included the year of publication, the sector and the region affiliated with the research institution, the area of interest, the methodology, the number of authors, funding, collaboration, and the publishing journal. All variables were obtained from published manuscripts. A five-year interval from the year of publication was factored in. Collaboration was defined as institutional participation in data collection. Authors' affiliations with different institutions were considered a collaboration in meta-analyses, systematic reviews, and review articles only. Funded publications were identified by the information on funding grants in their manuscripts. The areas of interest of the publications were classified into 10 categories: abdomen, gastroenterology, trauma, thoracic, oncology, vascular, obesity, head and neck, medical education, and miscellaneous. 

As a measurement of research impact, the number of citations was obtained from Google Scholar, WoS, or Scopus. If there was an inconsistency in the number of citations between different databases, the highest number of citations was used. The age-corrected citation rate was calculated by dividing the number of citations by the number of years since the publication year [8].

The data were collected and managed using Microsoft Excel (2016). IBM SPSS Statistics for Windows (version 22) (IBM Corp., Armonk, NY) was used for data description and data analysis. The categorical variables, including year of publication, five-year interval from year of publication, research institution, affiliation, area of interest, methodology, funding, collaboration, and the publishing journal, are presented as frequencies. Numerical variables - including the number of authors, citation, and age-corrected citation - were described in median and/or ranges. Chi-squared test was used to compare the outcome variable groups and all categorical variables, and a t-test in numerical variables. A p-value <0.05 was considered statistically significant.

## Results

**Figure 1:**
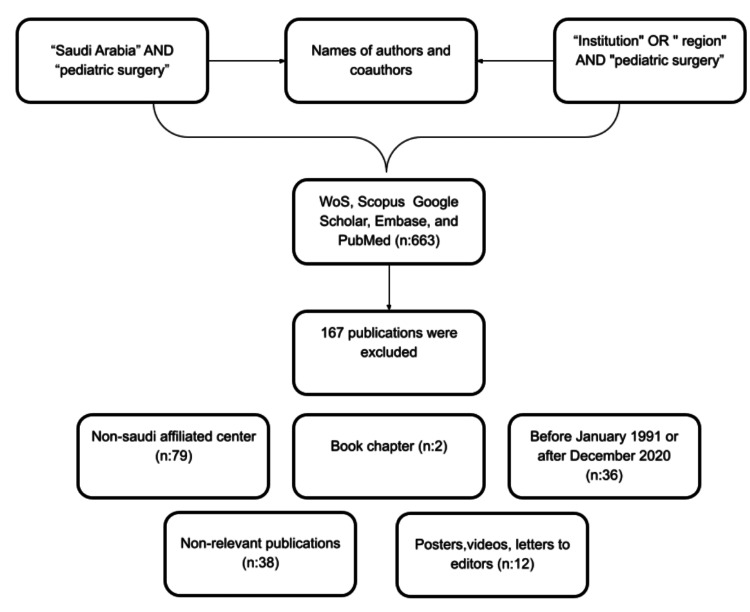
Chart depicting the data collection process

The total number of reviewed publications was 663; only 496 publications met the inclusion criteria. The reasons for exclusion are demonstrated in Figure 1. The total number of publications per year has increased gradually over the years (p<0.001). The number of publications in 1991 was seven, compared with 28 publications in 2020. The highest number of publications per year was 35 in 2014, while the lowest number was two in 1993. Over the five-year interval period, the highest cumulative publications were recorded between 2016 and 2020, with 131 publications, compared with 28 publications between 1991 and 1995 (Table 1).

**Table 1:** Number of publications per five-year intervals

Year of publication	N (%)
1991 - 1995	28 (5.6)
1996 - 2000	59 (11.9)
2001 - 2005	53 (10.7)
2006 - 2010	95 (19.2)
2011 - 2015	130 (26.2)
2016 - 2020	131 (26.4)
Total	496 (100)

The highest number of publications was from the Ministry of Health (MOH), the Ministry of Education (MOE), and the Ministry of National Guard (MNG), with 166, 160, and 66 publications, respectively. These sectors' publication productivity was the highest between the years 2011 - 2015. The private sector started to contribute to Saudi pediatric surgery literature in 2013, with eight publications from 2013 to 2020 (Table 2).

**Table 2:** Number of publications per sector over five-year intervals Data are presented as n (%)

Sector/year	1991 - 1995	1996 - 2000	2001 - 2005	2006 - 2010	2011 - 2015	2016 - 2020	Total
Ministry of Health	6 (3.6)	18 (10.8)	23 (13.9)	40 (24.1)	43 (25.9)	36 (21.7)	166 (33.5)
Ministry of Education	12 (7.5)	18 (11.3)	14 (8.8)	31 (19.4)	45 (28.1)	40 (25)	160 ( 32.3)
National Guard	0 (0)	9 (13.6)	9 (13.6)	6 (9.1)	26 (39.5)	16 (24.2)	66 (13.3)
King Faisal Specialist Hospital	10 (16.4)	11 (18)	1 (1.6)	9 (14.8)	9 (14.8)	21 (34.4)	61 (12.3)
Ministry of Defense	0 (0)	3 (8.6)	6 (17.1)	9 (25.7)	6 (17.2)	11 (31.4)	35 (7.1)
Private sector	0 (0)	0 (0)	0 (0)	0 (0)	1 (12.5)	7 (87.5)	8 (1.6)

In terms of region, 486 publications (98%) were affiliated with institutions in four of the 13 regions in Saudi Arabia. Publications related to pediatric surgery were the highest in the Riyadh Region, followed by the Makkah Region, the Eastern Region, and then the Asir Region. The contributions of the other regions in terms of publication productivity amounted to 10 publications: from Madinah (n = 4), Tabuk and Hail (n = 2), and Najran regions (n = 1) (Table 3).

**Table 3:** Publications per region over five-year intervals Data are presented as n (%)

Sector/year	1991 - 1995	1996 - 2000	2001 - 2005	2006 - 2010	2011 - 2015	2016 - 2020	Total
Riyadh	23 (9.5)	38 (15.8)	22 (9.2)	42 (17.5)	67 (28)	48 (20)	240 (48.4)
Makkah	5 ( 4.1)	9 (7.5)	10 (8.3)	20 (16.7)	23 (19.2)	53 (44.2)	120 ( 24.2)
Eastern Province	0 (0)	10 (9.5)	18 (17.1)	26 (24.8)	36 (34.3)	15 (14.3)	105 (21.2)
Asir	0 (0)	2 (9.6)	3 (14.3)	7 (33.3)	2 (9.5)	7 (33.3)	21 (4.2)
Other regions	0 (0)	0 (0)	0 (0)	0 (0)	2 (20)	8 (80)	10 (2)

Table 4 illustrates the number of publications based on the area of interest of the publications. The publications were categorized into 10 categories. The publications that included abdomen and gastroenterology were the most common, followed by genitourinary publications and thoracic and pulmonary publications. Nine publications were related to the field of medical education, and 25 publications fell under miscellaneous publications, including congenital syndromes, basic sciences, conjoined twins, robotics surgeries, child abuse, and transplant surgery.

**Table 4:** Publications by area of interest

Area of interest	N (%)
Abdomen and gastroenterology	225 (45.4)
Genitourinary	82 (16.5)
Thoracic and pulmonary	59 (11.9)
Oncology and hematology	33 (6.7)
Miscellaneous	25 (5)
Trauma	18 (3.6)
Obesity	17 (3.4)
Vascular	15 (3)
Head and neck	13 (2.6)
Medical education	9 (1.8)
Total	496 (100)

The publications were divided into 10 categories based on methodology. Case reports and case series constituted the highest number of publications, representing 49.4% of the total publications, and ranged from 44.2% to 62.7% of the total publications in each five-year interval, followed by retrospective chart reviews, which represented 30.2% and ranged from 19.8% to 45.3% of the total publications in each five-year interval. Cohort studies were the third most common methodology and represented 8.7% of the total publications, ranging from 3.6% of publications between 1991 and 1995 to 16% of publications between 2016 and 2020. The total proportion of meta-analyses and systematic reviews was 1.4%, and that of clinical trials was 2.6% of publications. Table 5 demonstrates the number of publications by methodology over five-year intervals.

**Table 5:** Publications by methodology

Methodology	1991 - 1995	1996 - 2000	2001 - 2005	2006 - 2010	2011 - 2015	2016 - 2020	Total
	N (%) of publications methodology over 5-year intervals						
Case reports and case series	15 (53.5)	37 (62.7)	32 (60.3)	42 (44.2)	60 (46.2)	59 (45)	245 (49.4)
Retrospective chart review	10 (35.7)	15 (25.4)	18 (34)	43 (45.3)	38 (29.2)	26 (19.8)	150 (30.2)
Cohort studies	1 (3.6)	5 (8.5)	2 (3.8)	4 (4.2)	10 (7.7)	21 (16)	43 (8.7)
Cross-sectional studies	0 (0)	0 (0)	0 (0)	1 (1.1)	5 (3.8)	9 (6.9)	15 (3)
Clinical trials	0 (0)	1 (1.7)	1 (1.9)	3 (3.1)	1 (0.8)	7 (5.3)	13 (2.6)
Review articles	1 (3.6)	1 (1.7)	0 (0)	0 (0)	6 (4.6)	3 (2.3)	11 (2.2)
Case-control studies	1 (3.6)	0 (0)	0 (0)	0 (0)	6 (4.6)	1 (0.8)	8 (1.6)
Systematic review and meta-analysis	0 (0)	0 (0)	0 (0)	2 (2.1)	1 (0.8)	4 (3.1)	7 (1.4)
Animal and cell studies	0 (0)	0 (0)	0 (0)	0 (0)	2 (1.5)	0 (0)	2 (0.4)
Clinical practice guideline	0 (0)	0 (0)	0 (0)	0 (0)	1 (0.8)	1 (0.8)	2 (0.4)
Total	28 (5.6)	59 (11.9)	53 (10.7)	95 (19.2)	130 (26.2)	131 (26.4)	496 (100)

The number of authors ranged from a single author to 25 authors per publication, and the median number of authors was three per publication. There was no statistically significant difference between the number of authors with different publication methodologies (p = 0.57). The median number of authors in clinical trials, systematic reviews, and meta-analyses was less than or equal to the median number of authors in case reports, case series, and retrospective chart reviews (Table 6).

**Table 6:** Median number of authors by publication methodology

Methodology	Median	Range (minimum - maximum )
Clinical practice guideline	14	23 (2 - 25)
Case-control studies	7	8 (1 - 9)
Cross-sectional studies	6	10 (1 - 11)
Animal and cell studies	4	3 (2 - 5)
Retrospective chart review	4	3 (2 - 5)
Case reports and case series	3	10 (1 - 11)
Cohort studies	3	16 (1 - 17)
Clinical trials	3	9 (1 - 10)
Systematic review and meta-analysis	2	3 (1 - 4)
Review article	1	3 (1 - 4)
Total	3	24 (1 - 25)
P = O.57		

Collaboration was observed in 28 publications, representing 5.6% of the overall publications. Collaboration between centers in the same region was found in 14 publications, and collaboration between centers in different regions was found in 11 publications. Three publications were conducted through collaboration with centers in different countries: two collaborations with institutions in Egypt and one with an institution in Canada. The methodology included 13 retrospective chart reviews, five cohort studies, three randomized clinical trials, three case series, a case‒control study, a cross-sectional study, a review article, and a meta-analysis.

The majority of the publications did not receive any funds (n = 480, 96.3%), and only 16 publications (3.2%) stated that they had received funds. All of the funded publications were affiliated with MOE institutions and were conducted in Riyadh Region (n = 13), Makkah Region (n = 2), and Eastern Region (n = 1). The funded publications included seven retrospective chart reviews, two randomized clinical trials, two cross-sectional studies, two cohort studies, a clinical practice guideline, a case series, and an animal laboratory study.

The number of citations per publication ranged between 0 and 179 citations, and the median number of citations was six. Table 7 shows the median number of citations per publication in each five-year interval. The age-corrected citation rate ranged between 0 and 19.89, with a median of 0.61. Similar to the number of citations, the highest age-corrected citation rate occurred between 2006 to 2010, with a median of one citation per year (range: 0-7.64). Only one publication among the top 10 age-corrected citation rate cited publications was conducted in collaboration, and another was funded. All nine highly cited publications were retrospective chart reviews, except for one meta-analysis. There was no significant difference between the age-corrected citation rate and funded publications (p = 0.075). On the other hand, publications involving collaborations between institutions had a significantly higher age-corrected citation rate (p = 0.004), but the collaboration did not affect the number of citations (p = 0.67).

**Table 7:** Number of citations and age-corrected citation rate per publication over five-year intervals

Year interval	Number of citations, median (minimum - maximum)	Age-corrected citations rate, median (minimum - maximum)
1991 - 1995	8 (0 - 60)	0.28 (0 - 2.31)
1996 - 2000	11 (0 - 78)	0.52 (0 - 3.55)
2001 - 2005	8 (0 - 114)	0.4 (0 - 7.13)
2006 - 2010	13 (0 - 107)	1 (0 - 7.64)
2011 - 2015	7 (0 - 179)	0.87 (0 - 19.89)
2016 - 2020	1 (0 - 53)	0.33 (0 - 10.6)
Total	6 (0 - 179)	0.61 (0 - 19.89)

Only one publication within the top 10 age-corrected cation rate involved a collaboration with other institutions, and another was funded. In addition to one meta-analysis, all nine highly cited publications were retrospective chart reviews. There was no statistically significant difference between funded publications and the number of citations (p = 0.45) and the age-corrected citation rate (p = 0.075). Publications involving collaborations between institutions had a significantly higher rate of age-corrected citation rate (p = 0.004), but no difference was noted in the number of citations (p = 0.67).

The journals varied greatly in terms of international and national publishers. Table 8 shows journals with the highest number of Saudi publications.

**Table 8:** Top 10 journals with most publications

Journal	N (%)
Annals of Saudi Medicine	64 (12.9 )
Journal of Pediatric Surgery	56 (11.3 )
Pediatric Surgery International	39 (7.9 )
Saudi Medical Journal	36 (7.3 )
Annals of Pediatric Surgery	32 (6.5 )
Journal of Pediatric Surgery Case Reports	25 (5 )
European Journal of Pediatric Surgery	23 (4.6 )
Journal of King Abdulaziz University - Medical Science	13 (2.6 )
African Journal of Pediatric Surgery	11 (2.2 )
Others journals	197 (39.7)

## Discussion

This study is the first one to apply bibliometric analysis to pediatric surgery publications in Saudi Arabia. Pediatric surgery as a specialty is relatively new compared to other medical specialties in Saudi Arabia. The number of institutions providing pediatric surgery services and the number of pediatric surgeons have increased gradually over the last 40 years [10,11]. The number of publications in 1991 was 7; since then, the number of publications has increased 4-fold. This gradual growth in publication productivity can be explained by the increasing number of pediatric surgeons and institutions providing pediatric surgery services and the implementation of policies that encourage publications.

​The highest number of publications was conducted in institutions affiliated with the MOH, which can be attributed to the number of institutions compared with other sectors [11]. The second-highest number of publications was conducted in institutions affiliated with the MOE, which can be attributed to the nature of the academic field and academic surgeons. However, no governmental sector experienced a steady increase in the number of publications over the years. Many factors may contribute to the differences in the productivity of publications across sectors, including financial privileges, career advancement, and the presence of research centers. The number of publications from the Riyadh Region was greater than that from other regions and constituted almost half of the total number of publications. This region was followed by the Makkah, Eastern, and Asir regions. This can be attributed to the fact that most pediatric surgery centers and pediatric surgery tertiary centers are in those three regions. 

One of the characteristics of pediatric surgery as a specialty is its involvement in different body systems and diseases in a pediatric surgeon's clinical practice; this is represented in the variations in the publications' areas of interest. The highest number of publications involved the abdomen and gastroenterology, followed by genitourinary publications and thoracic publications. The publications on obesity are almost exclusively affiliated with the obesity research center at King Saud University in Riyadh (n=13), which is funded exclusively by the Obesity Research Chair. Moreover, the publication with the highest citation and age-corrected citation rates was funded by this research chair, demonstrating the efficiency of specialized research chairs in promoting high-quality research.

Trauma-related publications accounted for 3.6% of the total, a relatively low percentage given the prevalence and significance of road traffic accidents in Saudi Arabia. Traffic accidents are the second leading cause of death overall and the main cause of death in children [25]. Overall, there were only four publications related to road traffic accidents; the low number of publications on trauma might be attributed to the lack of trauma registries, and that children above the age of 14 years are treated by general surgeons. Publications in oncology represented only 6.7% of the total publications, and the majority of the publications were case reports and case series despite the presence of specialized oncology centers. This finding suggests that the area of pediatric surgical oncology remains underexplored in Saudi Arabia.

Research methodology is considered one of the most important parameters for critically appraising a publication. In our study, the most common methodologies used were case reports and case series. Case reports and case series are important descriptive tools for novel, unusual, or atypical cases and are important for educational purposes or for generating further research questions. They are especially attractive to researchers with limited resources and funding since they are feasible and easily written; unfortunately, they do not rank highly in the hierarchy of research evidence. Retrospective chart reviews represented 30.25% of the publications and were the second most commonly used methodology. Like case studies, retrospective chart methodologies require limited resources. This is followed by cohort studies, which have become the third most common methodology, as the percentage of cohort studies over 5-year intervals has increased over time.

Retrospective chart review publications increased gradually and peaked between 2006-2010 at 45.3% of publications; then, they started to decrease gradually until they reached 19.8% of publications between 2016-2020. During the same period, an increase in the number of cohort studies and cross-sectional studies was observed. The first published clinical trial was in 1998, whereas the first randomized controlled clinical trial was in 2014. The first systematic review was published in 2010, followed by the first meta-analysis in 2011. The number of systematic reviews and meta-analyses doubled between 2016 and 2020 compared with 2006-2010. There were two publications on animal and cell studies conducted in research labs, and there were two clinical practice guidelines. Notably, the methodologies that ranked higher in the hierarchy of evidence have been utilized more in recent years than in older publications, which reflects the gradual evolution in the conduct of research.

Many factors contribute to the number of authors per publication, including the expected workload, human resources, financial support, and publishers’ authors' guidelines. Although the median number of authors across different methodologies did not differ significantly, our observation was that the number of authors did not match the expected number of authors according to methodology; for example, we observed controlled clinical trials and meta-analyses that were conducted by a single author, whereas 61 case reports and case series were conducted by five or more authors. The possibility of the presence of nonscientific factors, such as social or political factors, must be studied. On the other hand, the production of publications that are expected to need a greater number of research personnel with much smaller teams or a single author raises concerns about the quality and integrity of the publication. Further analysis of the publications’ authorship must be conducted to analyze the current situation and improve the quality of the literature.

Research funding is considered one of the major impetuses contributing to the successful execution of a research project; it provides research projects with the necessary materials and affords human resources when required [26]. While grant funds might not be required for some research, it is essential for other research projects [26]. In our study, we found that only 16 publications received a grant for their project. The number might be under-reported as the funding received in manuscripts is subject to publisher guidelines. All funded publications were affiliated with the MOE, despite the presence of grant committees in other sectors. Factors that might affect the granting of funds include the paucity of grant committees, funding agents, or research chairs related to pediatric surgery. In addition, difficulty in the application process or a lack of projects that require funds. The lack of funds may be one of the main reasons that the number of case reports, case series, and retrospective chart reviews is greater than other methodologies.

To improve the quantity and quality of national research productivity in pediatric surgery, we recommend studying the factors that affect research funding and the impact of funding on publication quality. Research collaboration between researchers and institutions has been widely recognized as essential in the literature, as collaboration enhances the quantity and quality of publications [27,28]. In our study, collaboration between centers was reported in 28 articles. Multiple factors contribute to a low number of collaborations, including the different patient data management systems utilized across different institutions and the lack of detailed censuses or national registries specialized in pediatric surgery. Other factors can include logistical challenges and the absence of policies that encourage collaboration.

The number of citations is considered one of the most important quality indicators of a publication and might represent peers' recognition [29]. The number of citations might be affected by paper-related factors, journal-related factors, and author-related factors [30]. In our study, the median number of citations was eight and ranged between 0 and 60 citations per publication between 1991 and 1995; it peaked between 2006 and 2010, as the median number of citations was 13 and ranged between zero and 107 citations, whereas the most cited publication was published in 2012. In our study, neither collaboration nor funding influenced the number of citations.

The age-corrected citation rate is another performance indicator that can be used to assess the quality of a publication [31]. The age-corrected citation rate over the 5-year interval revealed a curve comparable to the number of citations over the same period. The period between 2006 and 2010 had the highest age-corrected citation rate, with a median of one citation per year. We recommend that publications from that period be analyzed to further identify researchers, manuscripts, and journal-related factors that contributed to the increase in the number of citations and the increase in the rate of age-corrected citations. Indicators of the quality and structure of publications include the publication's journal. Among the 496 articles, 299 (60.3%) were published in nine journals. Three journals were local journals that were more interested in all Saudi Arabia-related papers than just pediatric surgery publications, whereas six journals were prestigious worldwide journals focusing on pediatric surgery. The journals' content reflects the caliber of Saudi Arabian publications on pediatric surgery.

Limitations

This study has several limitations that should be acknowledged. First, as the first bibliometric analysis focusing on pediatric surgery publications in Saudi Arabia, it may not comprehensively capture all relevant research outputs-particularly those published in non-indexed local journals-potentially leading to underreporting. Second, data collection was based on author names and institutional affiliations, which may result in the overrepresentation of certain sectors or regions due to a snowball effect. Additionally, the analysis of authorship and funding may be affected by underreporting or misreporting, given the variability in publisher requirements and manuscript disclosures. Lastly, key factors influencing research productivity-such as institutional policies, resource availability, and individual motivation-were not directly assessed, limiting the ability to fully understand the drivers behind publication trends. Future studies incorporating qualitative methods, such as stakeholder interviews and broader data sources, could offer deeper insights and help advance pediatric surgical research in Saudi Arabia.

## Conclusions

This study demonstrated the gradual increase in publications related to pediatric surgery in Saudi Arabia over the last three decades. Most publications were not funded, and collaboration between institutions was very limited, although collaboration is correlated with a higher age-corrected citation rate. The number of citations and the age-corrected citation rate have declined after peaking between 2006 and 2010, despite the growing number of publications. To improve the quantity and quality of national research productivity in pediatric surgery, we recommend the employment of research-specialized human resources, the creation of a data registry, and the implementation of policies to support collaboration between different centers. Further analysis of the factors affecting sector and regional productivity is also recommended.
